# Evaluation of Soluble Junctional Adhesion Molecule-A as a Biomarker of Human Brain Endothelial Barrier Breakdown

**DOI:** 10.1371/journal.pone.0013568

**Published:** 2010-10-21

**Authors:** Axel Haarmann, Annika Deiß, Jürgen Prochaska, Christian Foerch, Babette Weksler, Ignacio Romero, Pierre-Olivier Couraud, Guido Stoll, Peter Rieckmann, Mathias Buttmann

**Affiliations:** 1 Department of Neurology, Julius Maximilian University, Würzburg, Germany; 2 Department of Neurology, University of Frankfurt, Frankfurt, Germany; 3 Divison of Hematology-Medical Oncology, Weill Medical College of Cornell University, New York, New York, United States of America; 4 Department of Biological Sciences, The Open University, Milton Keynes, United Kingdom; 5 Institut Cochin, Université Paris Descartes, Paris, France; City of Hope National Medical Center, United States of America

## Abstract

**Background:**

An inducible release of soluble junctional adhesion molecule-A (sJAM-A) under pro-inflammatory conditions was described in cultured non-CNS endothelial cells (EC) and increased sJAM-A serum levels were found to indicate inflammation in non-CNS vascular beds. Here we studied the regulation of JAM-A expression in cultured brain EC and evaluated sJAM-A as a serum biomarker of blood-brain barrier (BBB) function.

**Methodology/Principal Findings:**

As previously reported in non-CNS EC types, pro-inflammatory stimulation of primary or immortalized (hCMEC/D3) human brain microvascular EC (HBMEC) induced a redistribution of cell-bound JAM-A on the cell surface away from tight junctions, along with a dissociation from the cytoskeleton. This was paralleled by reduced immunocytochemical staining of occludin and zonula occludens-1 as well as by increased paracellular permeability for dextran 3000. Both a self-developed ELISA test and Western blot analysis detected a constitutive sJAM-A release by HBMEC into culture supernatants, which importantly was unaffected by pro-inflammatory or hypoxia/reoxygenation challenge. Accordingly, serum levels of sJAM-A were unaltered in 14 patients with clinically active multiple sclerosis compared to 45 stable patients and remained unchanged in 13 patients with acute ischemic non-small vessel stroke over time.

**Conclusion:**

Soluble JAM-A was not suited as a biomarker of BBB breakdown in our hands. The unexpected non-inducibility of sJAM-A release at the human BBB might contribute to a particular resistance of brain EC to inflammatory stimuli, protecting the CNS compartment.

## Introduction

Junctional adhesion molecule-A (JAM-A) is a member of the immunoglobulin superfamily. It is expressed in endothelial and epithelial tight junctions, by mononuclear cells, neutrophils and thrombocytes [1], [2], [3], [4]. In endothelial and epithelial cells, JAM-A contributes to the paracellular solute barrier by formation of JAM-A homodimers within the cell membrane (*in cis*), binding homodimers of neighbouring cells (*in trans*) [5], [6], [7], [8], [9]. Upon inflammatory stimulation of endothelial cells (EC), JAM-A redistributes from tight junctions to the apical cell surface where it mediates immune cell adhesion *in vitro* under static and flow conditions [10], [11], [12], [13], [14]. Chemokine-triggered leukocyte transmigration across cultured EC is also mediated by endothelial JAM-A, however without endothelial JAM-A redistribution as a prerequisite [11]. T-cell and neutrophil adhesion and transmigration governed by endothelial JAM-A were found to be mediated by αLβ2-integrin (Leukocyte Function Antigen-1, LFA-1) on leukocytes [11], a result that could not be confirmed by another group [15]. In line with the *in vitro* evidence highlighting the importance of endothelial JAM-A for immune cell extravasation, *ex vivo* perfusion of wire-injured carotid arteries from JAM-A^−/−^ apoE^−/−^ mice with MM6 monocytic cells demonstrated reduced MM6 extravasation in comparison to arteries from JAM-A^+/+^ mice [16]. Neutrophil extravasation *in vivo* in mice lacking endothelial JAM-A was found to be reduced in a model of ischemia reperfusion injury of the liver [17] but not of the heart [18]. Together these studies indicated a tissue-specific role of endothelial JAM-A in the regulation of leukocyte extravasation.

A soluble form of JAM-A (sJAM-A) can be detected in the peripheral blood [19]. Increased sJAM-A plasma or serum levels compared to healthy controls were described in patients with coronary artery disease, arterial hypertension, systemic sclerosis, and renal insufficiency undergoing hemodialysis [19], [20], [21], [22]. Two different groups recently identified human EC, namely dermal microvascular EC (HDMEC) and umbilical vein EC (HUVEC), as a cellular source of sJAM-A. Both EC types showed a constitutive release of sJAM-A into culture supernatants which was enhanced by pro-inflammatory stimulation [22], [23]. These *in vitro* data supported the blood level studies suggesting sJAM-A as a biomarker of vascular inflammation. In HUVEC, sJAM-A was found to be shedded from the cell surface by the disintegrin and metalloproteinases (ADAM) 10 and 17 upon pro-inflammatory stimulation [23]. On a functional level, recombinant sJAM-A reduced adhesion of mononuclear cells to cultured HUVEC and HDMEC [22], [24]. Furthermore it reduced chemokine-triggered endothelial transmigration of CD4^+^ CD45RO^+^ memory T cells across HUVEC under static and flow conditions [24]. Finally, recombinant sJAM-A inhibited neutrophil extravasation *in vivo* in an air pouch model of vascular inflammation [23]. Based on these results, it was suggested that sJAM-A might limit leukocyte extravasation at sites of vascular inflammation. However, it was also noted that all published experiments demonstrating a reduction of leukocyte extravasation by recombinant sJAM-A used concentrations too high to allow final conclusions about the pathophysiological function of sJAM-A *in vivo* [23]. To date, the *in vivo* function of sJAM-A has not finally been addressed.

To protect CNS homeostasis, EC forming the blood-brain barrier (BBB) are highly specialized and differ in many molecular aspects from other EC types such as HUVEC [25], [26]. A potential role of JAM-A at the BBB under inflammatory conditions was indicated by a study reporting reduced brain endothelial JAM-A immune staining in the rat cortical cold injury model [27]. Reduced endothelial JAM-A immune staining was also observed in active brain lesions of patients with multiple sclerosis (MS) [28], [29]. No major difference of brain EC JAM-A expression levels was noted between mice with cytokine-induced meningitis and sham-treated animals [30]. The expression of sJAM-A at the BBB has not been investigated so far.

Here we studied the regulation of JAM-A expression in cultured human brain microvascular EC (HBMEC) and evaluated sJAM-A as a serum marker of BBB breakdown. While inflammatory stimulation of cultured HBMEC induced a subcellular redistribution of cell-bound JAM-A as previously described in other EC types, a detected constitutive release of sJAM-A was unexpectedly neither enhanced by pro-inflammatory nor by hypoxia/reoxygenation challenge. Accordingly, sJAM-A serum levels did not indicate a BBB breakdown *in vivo*. These results define a novel feature of human brain EC, distinguishing them from other EC types and possibly contributing to their particular anergy in the protection of CNS homeostasis.

## Materials and Methods

### Cell Culture

Cryopreserved single donor primary HBMEC isolated from normal human brain were purchased from Cell Systems (Kirkland, WA) at passage 2. Each preparation was extensively characterized as previously described [31]. Cells were plated on 2% gelatin-coated culture dishes or flasks of various sizes depending on the experiment (all from Nunc, Roskilde, Denmark) and cultured at 37°C, 5% CO_2_ in M199 medium containing 10% fetal calf serum, 20 µg/mL endothelial cell growth supplement (20 µg/mL; Sigma-Aldrich, Deisenhofen, Germany), 100 µg/mL heparin, 250 µg/mL amphotericin B, 50 µg/mL gentamycin, 50 U/mL penicillin and 50 µg/mL streptomycin. HBMEC were used at passage 4 and 5 for experiments. A well characterized immortalized HBMEC line, hCMEC/D3, was cultured as previously described [32]. For hypoxia experiments, EC were cultured in a C60 incubator from Labotect (Göttingen, Germany).

### Immunocytochemistry

For immunocytochemistry, cells were either fixed with 3.7% paraformaldehyde (PFA) for 10 min and permeabilized with 0.1% Triton X-100 for 6 min at room temperature (JAM-A, ZO-1) or fixed with a fresh 1∶1 mixture of methanol and acetone for 10 min at −20°C (occludin), and then washed with washing buffer containing 0.2% gelatin in Dulbecco's phosphate-buffered saline. Subsequently, primary mouse monoclonal antibodies against JAM-A (clone M.Ab.F11, AbD Serotec, Düsseldorf, Germany), zonula occludens-1 (ZO-1, clone ZO-1-1A12, Zymed, San Francisco, CA) or occludin (clone OC-3F10, Zymed) were incubated at 4°C overnight. Subsequently, a Cy3-coupled anti-mouse secondary antibody was incubated for 1 h at room temperature. F-actin staining was performed with Alexa Fluor 488 phalloidin according to the instructions of the manufacturer (Invitrogen, Karlsruhe, Germany). Nuclei were counterstained with DAPI. Finally, an antifading agent (Dabco, Merck, Germany) was added. Negative controls were performed by omitting the primary antibodies as well as by staining with isotype-matched control antibodies. The stainings were analyzed by an Olympus IX-70 inverted system microscope with IX-FLA observation attachment for fluorescence imaging.

### Dextran Permeability Assay

To assess the paracellular permeability of HBMEC for dextran 3000 a transwell system was used. 1×10^5^/well HBMEC in 200 µL medium were seeded onto polycarbonate membrane inserts (pore size 0.4 µm, surface area 0.33 cm^2^) purchased from Corning Inc. (Cat. no. 3413, Corning, NY). After reaching confluence (as confirmed by DAPI staining of filters grown in parallel), cells were left untreated or stimulated as indicated for 24 hours. Then the cell culture medium was replaced by sterile-filtered, prewarmed (37°C) 10 mM HEPES buffer, pH 7.2, containing 0.1% bovine serum albumin and 4.5% (w/v) glucose. After equilibration of the cells for 5 min at 37°C, 5% CO_2_ FITC-dextran 3000 (Sigma-Aldrich) was added to the upper chambers in a volume of 4 µL and to a final concentration of 1 mg/mL, and incubated at 37°C, 5% CO_2_ for 90 min. Thereafter relative fluorescence in the lower chambers was determined using a Fluoroskan Ascent® (Thermo Electron Corporation, Dreieich, Germany) microplate fluorometer at an excitation wavelength of 485 nm and an emission wavelength of 538 nm. All experiments were performed in octuplicates. FITC-dextran clearance through coated and uncoated filters with no cells attached served as additional control conditions.

### Flow Cytometry

Resting and stimulated EC were harvested and dissociated with Accutase™ (Sigma-Aldrich), a mixture of proteolytic and collagenolytic enzymes from an invertebrate species. Single cell suspensions were then stained according to a standard protocol as previously described [31] using M.Ab.F11, rabbit anti-human JAM-A (Cat. no. 36–1700, Zymed), or fluorescein-coupled monoclonal mouse anti-human intercellular adhesion molecule-1 (ICAM-1, clone BBIG-I1, R&D Systems, Karlsruhe, Germany) and appropriate isotype controls as primary antibodies, and analyzed using a Beckton-Dickinson (Mountain View, CA) fluorescence-activated cell sorter.

### Western Blotting

For generation of whole cell protein extracts, cells grown to subconfluency in 25 cm^2^ flasks were stimulated as indicated, washed with icecold PBS and scraped into radioimmunoprecipitation assay (RIPA) buffer composed of 50 mmol/L Tris-HCl, pH 7.4; 150 mmol/L NaCl; 1% Nonidet P40; 0.25% sodium deoxycholate and 1× Roche COMPLETE® protease inhibitor mix (Roche Diagnostics GmbH, Mannheim, Germany). Samples were shaken vigorously for 30 minutes, and then centrifuged at 20,000 g for 20 minutes at 4°C. Supernatants were subjected to Western blot analysis.

For analysis of sJAM-A levels in cell culture supernatants 1.3 mL cell culture supernatant were transferred to an 1.5 mL Eppendorf tube. After addition of 35 µL sodium deoxycholate 10% and gentle mixing samples were incubated at room temperature for 10 minutes. Thereafter 150 µL trichchloroacetic acid 77% were added, samples were vortexed and incubated on ice for 30 minutes, and then centrifuged at 20,000 g for 20 minutes at 4°C. Then all supernatant was carefully removed, 300 µL ice-cold acetone were added and samples were centrifuged at 20,000 g for 5 minutes at 4°C. Thereafter the supernatant was carefully removed and the pellet was dried. After resuspension in equal amounts of loading buffer and heating at 65°C for 3 minutes samples were subjected to SDS-PAGE.

M.Ab.F11, rabbit anti-human JAM-A (Zymed) or goat anti-human JAM-A (Cat. no. AF-1103, R&D) were used as primary antibodies for JAM-A detection as indicated. Appropriate peroxidase-coupled secondary antibodies were employed with a standard enhanced chemoluminescence system (Amersham, Arlington Heights, IL, USA). After peroxidase inactivation, the same membranes were reprobed with a rabbit polyclonal or a mouse monoclonal antibody against β-actin (both from Sigma-Aldrich) as a loading control.

### Enzymatic N-Deglycosylation

N-deglycosylation was performed using the GlycoProfile™ II Enzymatic In-Solution N-Deglycosylation kit by Sigma-Aldrich according to the instructions of the manufacturer. In brief, 50 µg of a whole cell protein extract generated by the RIPA lysis buffer described in the previous section were denaturated by addition of n-octyl-D-glucopyranoside and 2-mercaptoethanol, and incubation at 100°C for 10 min. Then the samples were incubated with the indicated amounts of PNGase F in incubation buffer for 2 h. N-glycosylated recombinant RNase B was digested in parallel as a positive control. The reaction was stopped by heating at 100°C for 10 min. Finally, the samples were analyzed on Western blots.

### Enzyme-Linked Immunosorbent Assay

To detect sJAM-A we developed a sandwich ELISA employing NUNC Maxi Sorp 96-well plates and appropriate reagents from R&D Systems: goat anti-human JAM-A (Cat. no. AF1103) at 4°C overnight as capture antibody, biotinylated goat anti-human JAM-A (Cat. no. BAF 1103) at 37°C for 30 min as detection antibody, recombinant human JAM-1/Fc chimera (Cat. no. 1103-JM) for the standard curve which was run on every ELISA plate and the substrate reagent pack DY999. All samples were measured in duplicates. Furthermore all serum samples were measured undiluted, at 1∶20 and at 1∶400 dilution in parallel. The minimum detectable concentration defined as standard curve duplicate significantly exceeding the background signal was 1.526 ng/mL in all assays used for sample measurement. While JAM-A could be detected in all serum samples, JAM-A in cell culture supernatants could consistently be detected only after protein concentration by filter centrifugation, using Amicon Ultra-15 Ultracel-10k filter devices (Millipore Corporation, Tullagreen, Ireland). This procedure revealed a ∼10-fold JAM-A concentration as determined by filter centrifugation of dilutions of recombinant JAM-A in complete medium.

### Patients

The study was performed in accordance to the Declaration of Helsinki. It was approved by the local ethics committees. Written informed consent was given by all patients.

All MS patients were treated at the University of Würzburg, Department of Neurology, and had relapsing-remitting MS according to the McDonald criteria [33]. Clinical details are provided in online supplementary Table S1. None of the MS patients had received treatment with corticosteroids, interferon-β, glatiramer acetate, azathioprine, natalizumab or mitoxantrone during the 3 months preceding blood withdrawal for study participation, most often due to patient denial of secondary prophylactic pharmacotherapy. High-dose corticosteroid treatment in the relapse group was initiated after blood withdrawal for this study. Group assessment of MS patients (active vs. stable) was solely based on clinical parameters as gadolinium-enhanced MRI scans around the time point of blood withdrawal were available only for a minority of clinically active patients (n = 3) because high-dose steroid pulse therapy, which effectively suppresses gadolinium enhancement, was started at the day of admission in most patients with an acute relapse while MRI scans were not immediately available in most of these patients due to logistic reasons. Most stable patients were seen in an outpatient setting and had not received very recent MRI scans.

All patients with non-small vessel ischemic stroke were treated at the University of Frankfurt, Department of Neurology. Serum samples were collected within the framework of a prospective study on stroke biomarkers [34]. We selected all patients with complete ischemic stroke not undergoing hemorrhagic stroke transformation in the course of their disease, for whom serum samples both collected within the first 3 h after clinical onset of symptoms and 24 h thereafter were available (n = 13). See online supplementary Table S2 for clinical details.

### Statistics

Levels of sJAM in HBMEC culture supernatants were analyzed by the Kruskal-Wallis test. To compare baseline characteristics of study participants we used the Mann-Whitney U test or the chi-square test. To analyze permeability assays or to compare JAM-A serum levels between MS patients with stable disease and other MS patients during an acute relapse the Mann-Whitney U test was employed. For comparison of serum levels in stroke patients over time we used the Wilcoxon matched pairs test. A *p* value <0.05 was considered statistically significant. All data were analyzed with GraphPad PRISM 4 software (GraphPad Software, La Jolla, CA).

## Results

### JAM-A in Cultured HBMEC Is Redistributed at the Subcellular Level upon Inflammatory Stimulation

**Figure 1:**
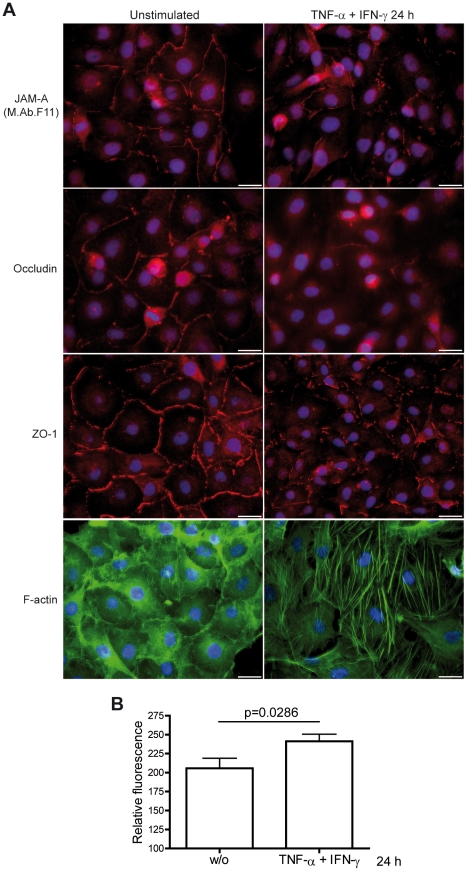
Immunocytochemical JAM-A tight junction staining in primary HBMEC is reduced under barrier-disturbing pro-inflammatory conditions. Cells were either left unstimulated or treated with TNF-α 10 ng/mL and IFN-γ 100 U/mL for 24 h. **A** Immunocytochemical staining was performed with primary antibodies against JAM-A (M.Ab.F11), occludin and ZO-1. F-Actin was stained with Alexa Fluor 488 phalloidin. Representative for 5 independent experiments with different HBMEC preparations. **B** Analysis of paracellular barrier function by dextran 3000 transwell permeability assays. Mean and SD of 4 independent experiments in octuplicates with different HBMEC preparations. The two-tailed Mann-Whitney U test was used for statistical analysis. Bar = 25 µm.

**Figure 2:**
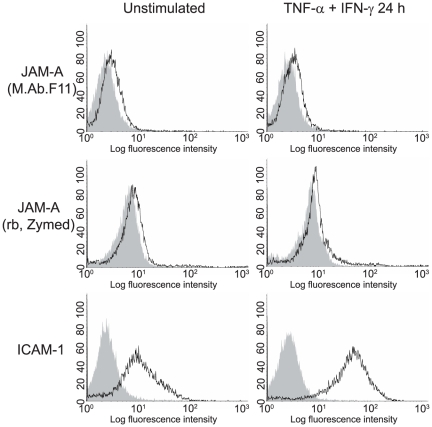
In contrast to ICAM-1, JAM-A overall surface expression levels on HBMEC do not change upon pro-inflammatory stimulation. Flow cytometric analysis of Accutase™-dissociated single cell suspensions of primary HBMEC after stimulation as in Figure 1. JAM-A was stained with M.Ab.F11 and a rabbit polyclonal antibody from Zymed. Staining against ICAM-1 served as a positive control. In parallel, unstimulated and stimulated HBMEC were stained with matched isotype control antibodies. Filled histograms represent isotype stainings, open histograms JAM-A and ICAM-1 stainings. Representative for 5 independent experiments with different HBMEC preparations.

**Figure 3:**
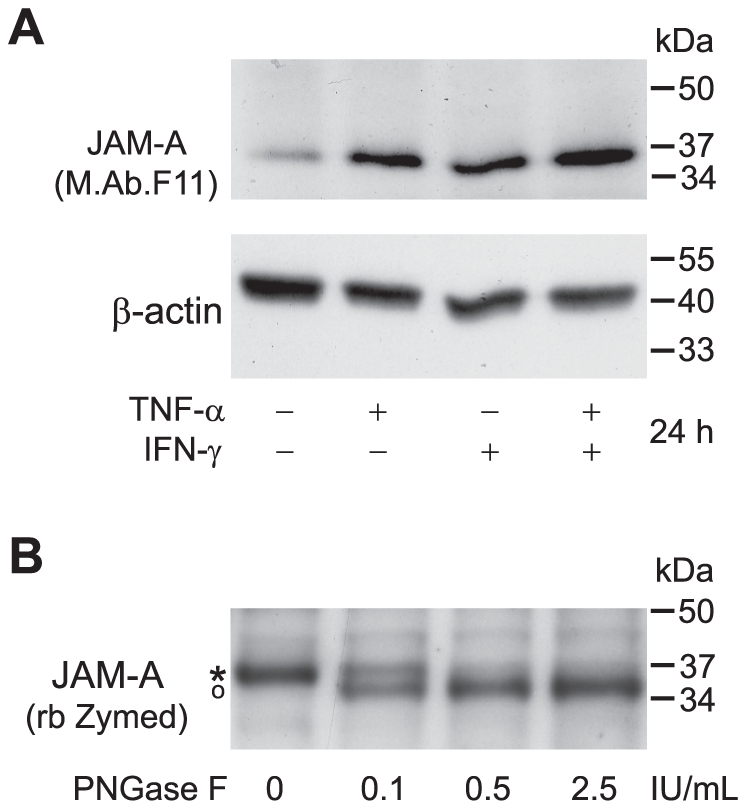
Pro-inflammatory stimulation of HBMEC induces JAM-A dissociation from the actin cytoskeleton. **A** Primary HBMEC were either left unstimulated or treated with TNF-α 10 ng/mL or IFN-γ 100 IU/mL alone or in combination. Cell protein extracts were generated with a Nonidet-P40 based cell lysis buffer and subjected to Western blot analysis. JAM-A was stained with M.Ab.F11. Staining of the same, peroxidase-inactivated membranes with a rabbit antibody against β-actin served as a loading control. **B** N-deglycosylation of JAM-A with increasing concentrations of PNGase F for 2 h. A rabbit polyclonal antibody against JAM-A (Zymed) was used for the detection of JAM-A. The asterisk represents N-glycosylated, the open circle N-declycosylated JAM-A. Representative experiments out of at least 5 independent experiments with different EC preparations for each subpanel of the figure are shown.

To investigate whether JAM-A is expressed in cultured primary HBMEC and to study its expression and subcellular localization under inflammatory conditions we first performed immunocytochemical staining of primary single donor HBMEC preparations employing M.Ab.F11 for JAM-A staining, the monoclonal antibody by which JAM-A was originally identified [1]. As observed for the transmembranous tight-junction protein occludin and the intracellular adapter protein ZO-1, JAM-A staining was linear and localized to tight junctions under resting conditions. The signal was reduced and more punctuate upon stimulation for 24 h with tumour necrosis factor-α (TNF-α) and interferon-γ (IFN-γ). In parallel we observed a reorganization of the actin cytoskeleton with formation of stress fibers (Figure 1A). Altered tight junction morphology under inflammatory conditions corresponded to an increased paracellular permeability for dextran 3000, as determined in transwell assays (Figure 1B). Flow cytometric analysis of Accutase™-dissociated single cell suspensions with two different antibodies against JAM-A demonstrated that overall JAM-A cell surface expression levels did not change upon inflammatory stimulation with TNF-α and IFN-γ while intercellular adhesion molecule-1 (ICAM-1), serving as a positive control, was upregulated on the cell surface (Figure 2). Western blot experiments with M.Ab.F11 as primary antibody showed that TNF-α and IFN-γ enhanced the Nonidet-P40 soluble fraction of JAM-A in a slightly additive manner, indicating JAM-A dissociation from the cytoskeleton (Figure 3A). In contrast to recent results in HUVEC where JAM-A was detected as a single band at 43 kDa, or 38 kDa after N-deglycosylation [23], JAM-A in HBMEC was detected as a single band at 35–37 kDa depending on the cell preparation. This corresponded to the JAM-A size of 37 kDa reported in hCMEC/D3 by Huang and colleagues [35], which was confirmed by our experiments using hCMEC/D3 (data not shown). After N-deglycosylation, JAM-A appeared as a uniform band at ∼35 kDa, indicating that the molecular weight variability of JAM-A in HBMEC was due to differential N-glycosylation (Figure 3B). In summary, our results indicate a redistribution of JAM-A away from tight junctions and dissociation from the actin cytoskeleton under pro-inflammatory conditions while overall cell surface expression levels do not change. Similar results were obtained when using the immortalized human brain EC line hCMEC/D3 (data not shown).

### Cultured HBMEC Constitutively Release sJAM-A, which Is not Altered under Inflammatory and Hypoxia/Reoxygenation Conditions

**Figure 4:**
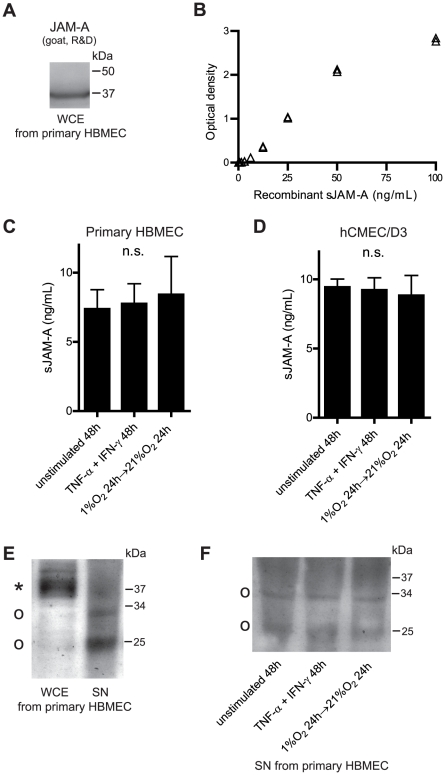
HBMEC constitutively release sJAM-A, which is not altered under inflammatory or hypoxia/reoxygenation conditions. **A** Representative Western blot of a HBMEC whole cell protein extract, employing the goat anti-JAM-A antibody which was used as capture antibody for a self-developed sandwich ELISA (compare Figure 3). **B** Typical standard curve of the self-developed ELISA for JAM-A detection in culture supernatants and serum. **C** and **D** Concentrations of sJAM-A in culture supernatants of primary HBMEC (**C**) or hMCEC/D3 (**D**) which were either left unstimulated for 48 h, stimulated with TNF-α 10 ng/mL and IFN-γ 100 U/mL for 48 h or subjected to 1% O_2_ for 24 h followed by a reoxygenation period of 24 h. Please note: cell culture supernatants were concentrated by filter centrifugation before ELISA measurement, resulting in ∼10-fold increased JAM-A concentration. Mean and SD of 3 independent experiments. The Kruskal-Wallis test was used for statistical analysis. n.s., not significant. **E** Comparative Western blot analysis of a whole cell protein extract and a TCA-precipitated protein extract from cell culture supernatant, using a rb polyclonal antibody (Zymed) for JAM-A detection. Representative of 3 independent experiments with different HBMEC preparations. The asterisk represents full-length JAM-A, open circles represent soluble JAM-A. **F** Comparative Western blot analysis of JAM-A in TCA-precipitated cell culture supernatants, using the same primary antibody as in E, after stimulation as in C and D. Representative of 3 independent experiments with different HBMEC preparations. Abbreviations: WCE, whole cell protein extract; SN, supernatant.

Having observed a similar inflammatory-induced alteration of cell-bound JAM-A expression as previously described in non-CNS EC types, we next investigated whether HBMEC show a basal or inducible release of sJAM-A as previously described in HUVEC and HDMEC [22], [23]. To measure sJAM-A, we developed a sandwich ELISA. When used for Western blot experiments with whole cell protein extracts, the goat anti-JAM-A antibody which was employed as capture antibody for this ELISA detected the same 35–37 kDa protein studied in Figure 1, 2, 3 (Figure 4A). Figure 4B shows a typical standard curve of the ELISA. We detected a basal release of sJAM into primary HBMEC (Figure 4C) and hCMEC/D3 (Figure 4D) culture supernatants which was neither altered by stimulation with TNF-α and IFN-γ for 48 h nor by exposure to 1% O_2_ for 24 h followed by a reoxygenation period of 24 h. Soluble JAM-A was not detected in complete medium without previous cell contact; concentrations increased over time of cell culture; and cell densities were not significantly influenced by stimulation conditions (data not shown). To confirm the key result of unaltered sJAM-A release during inflammatory or hypoxia/reoxygenation challenge by an alternative method, we additionally investigated sJAM-A levels in TCA-precipitated protein extracts from culture supernatants of primary HBMEC by Western blotting. A preceding comparative analysis of whole cell protein extracts and TCA-precipitated culture supernatants revealed, that sJAM-A had a smaller molecular weight than JAM-A in whole cell protein extracts. In supernatants, we detected two bands of ∼33 and ∼25 kDa (Figure 4E). Importantly, neither stimulation with TNF-α and IFN-γ for 48 h nor exposure to 1% O_2_ for 24 h followed by a reoxygenation period of 24 h altered the release of sJAM-A as detected by Western blotting which confirmed the results obtained by ELISA (Figure 4F). Serum starvation (2% FCS) prior to stimulation did not result in an inflammatory- or hypoxia/reoxygenation-inducible sJAM-A release from HBMEC either, as measured by ELISA (data not shown).

### Serum Levels of sJAM-A in Patients with Multiple Sclerosis and Ischemic Stroke Do not Reflect Disturbance of the Blood-Brain Barrier

**Figure 5:**
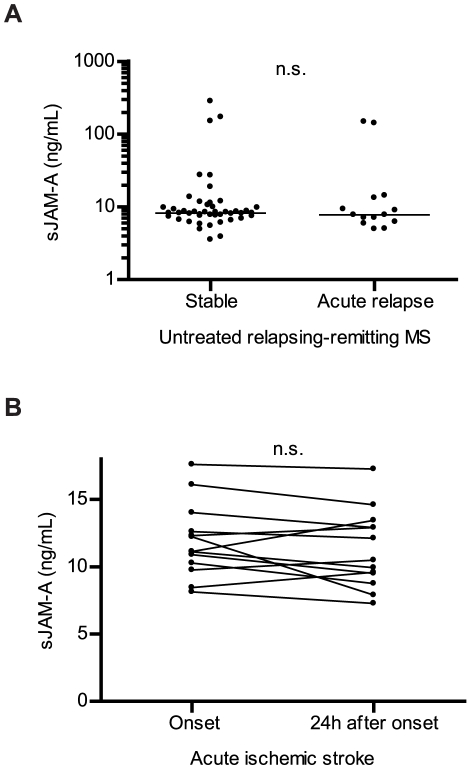
Soluble JAM-A serum levels do not indicate BBB disturbance in multiple sclerosis and ischemic stroke. **A** Levels of sJAM-A in serum samples from 45 patients with stable relapsing-remitting MS not receiving secondary prophylactic therapy were measured in duplicates by ELISA and compared to those in 14 untreated MS patients during an acute relapse. **B** Levels of sJAM-A were measured in serum samples from 13 patients with non-small vessel complete ischemic stroke where a first sample was obtained within the first 3 h of symptom onset and a second sample 24 h after clinical onset. Serum samples were not concentrated before ELISA measurement. Dots represent single values, lines median values. Serum levels in stable and inflammatory-active MS patients were statistically compared by the Mann-Whitney U test, serum levels in stroke patients over time by the Wilcoxon matched pairs test. n.s., not significant.

To investigate whether the release of sJAM-A from HBMEC *in vivo* is increased in disorders with disturbance of the BBB we first compared JAM-A serum levels in 45 clinically stable patients with relapsing-remitting MS not receiving immune therapy to serum levels in 14 active untreated MS patients not yet receiving steroid therapy during an acute relapse. Group assessments of MS patients were solely based on clinical parameters. No significant difference of sJAM-A serum levels was detected between both groups (Figure 5A). No specific clinical characteristics could be identified in 5 MS patients with high serum levels above 100 ng/mL. Next we investigated sJAM-A serum levels in 13 patients with acute, non-small vessel, complete ischemic stroke over time. A first set of serum samples was obtained within the first 3 h after clinical onset of symptoms when no significant alteration of tight junction molecule expression at the BBB is to be expected [36]. Another set of serum samples was obtained 24 h after onset of symptoms in a phase where commonly severe BBB disruption is observed in patients with complete ischemic stroke which was demonstrated in all stroke patients by repeated cranial imaging [37]. No significant alteration of sJAM-A serum levels over time in this group of patients was seen (Figure 5B). Our *in vitro* observations support the notion that soluble JAM-A is not inducibly released by HBMEC during inflammatory- or hypoxia/reoxygenation-induced BBB breakdown *in vivo*.

## Discussion

The major finding of this study is that in contrast to previously reported EC types, cultured human brain EC do not show an inducible release of sJAM-A under pro-inflammatory conditions, while the expression of cell-bound JAM-A upon pro-inflammatory stimulation is altered as previously described in other EC types. Accordingly, sJAM-A was not suited as a serum biomarker of human BBB breakdown in our hands.

The observed inducible loss of immunocytochemical JAM-A tight junction staining under pro-inflammatory conditions along with decreased staining of occludin and ZO-1 and a reorganization of the actin cytoskeleton, as well as an increase of the Nonidet-P40 soluble JAM-A fraction corresponds to previous results obtained with HUVEC and the murine heart microvascular EC line H5V, although the molecular weight of JAM-A is slightly higher in HUVEC compared to HBMEC [10], [12], [23]. This difference was found to be partially attributable to differential N-glycosylation of JAM-A in both cell types. Expression levels of cell-bound JAM-A on HBMEC as determined by flow cytometry and sJAM-A concentrations in cell culture supernatants from HBMEC under different culture conditions were found to be much lower than previously reported in HUVEC [19]. Differential JAM-A expression levels might therefore reflect cell-type specific differences. Our observation of subcellular JAM-A redistribution on HBMEC upon inflammatory stimulation with an overall reduction of immunocytochemical JAM-A staining adds *in vitro* evidence to *in situ* studies describing reduced immunohistochemical JAM-A staining at the BBB under inflammatory conditions [27], [29].

As recently reported for HUVEC and HDMEC [22], [23], we detected a constitutive release of sJAM-A from HBMEC into cell culture supernatants. However, in contrast to these cell types, the release of sJAM-A from HBMEC was not increased by pro-inflammatory stimulation with TNF-α and IFN-γ. This relative anergy of HBMEC in comparison to other EC types might reflect a particular resistance of HBMEC to inflammatory stimuli, protecting the CNS compartment. The release of sJAM from cultured HBMEC was not enhanced under hypoxia/reoxygenation conditions either, a condition that has not been studied in other EC types in the context of sJAM-A release so far. The parallel use of primary HBMEC and a well established immortalized HBMEC line, hCMEC/D3, for all *in vitro* experiments revealed comparable results, arguing for the robustness of our *in vitro* results.

Our *in vitro* results of a constitutive, but not an inducible, sJAM-A release by brain EC corresponded to our *in vivo* finding that sJAM-A serum levels in MS patients during an acute relapse and in patients with acute ischemic stroke remained stable. These results are in contrast to studies describing elevated sJAM-A serum levels in patients with inflammation in vascular compartments outside the brain [20], [21], [22]. Our *in vitro* results suggest that the stability of sJAM-A serum levels in patients with clinically active MS or ischemic stroke was rather due to a non-release at the BBB than to a masking effect by sJAM-A from other cellular sources. The detectable BBB disturbance in patients with a MS relapse might be subtle and focal, and our group assessment of MS patients was limited by the non-availability of MRI data, but ischemic stroke in a major territory, which was proven in all stroke patients by repeated cranial imaging, is always associated with a severe breakdown of the BBB 24 h after onset. Therefore, a release of sJAM-A, if present, should have been detectable in patients with stroke.

Based on our results it appears that sJAM-A is not suited as a serum biomarker for BBB breakdown in humans, which is in contrast to vascular cell adhesion molecule-1 (VCAM-1). Soluble VCAM-1 serum levels were reported to positively correlate in MS patients with clinical disease activity and an inflammatory BBB breakdown as indicated by Gadolinium-enhancing MRI lesions [38], [39]. Furthermore, enhanced serum levels of soluble VCAM-1 were reported in patients with ischemic stroke [40], [41]. Soluble VCAM-1 is released from HBMEC upon pro-inflammatory stimulation through a marimastat-inhibitable process [42], probably reflecting ADAM17 activation as demonstrated in murine EC [43]. JAM-A was found to be shedded from HUVEC by ADAM10 and 17 [23]. The cited studies in brain EC indicate that differences of inducible sJAM release under pro-inflammatory conditions between HBMEC and HUVEC are rather not due to differential ADAM17 activation in both cell types. The reason for a differential release of sJAM-A upon inflammatory stimulation of different EC types currently remains unknown.

Given that brain EC exhibit particularly strong barrier properties protecting the CNS, our results of a missing inducible sJAM-A release under pro-inflammatory conditions might somehow challenge the view that sJAM-A blocks leukocyte extravasation *in vivo* under pathophysiological conditions, as suggested by studies using recombinant JAM-A *in vitro* and *in vivo* [22], [23], [24]. Of note and in contrast to sJAM-A, the expression of cell-bound JAM-A in brain EC upon inflammatory stimulation *in vitro* is regulated as previously described in other EC types. It is well established that altered expression of cell-bound JAM-A under inflammatory conditions may facilitate leukocyte extravasation [10], [11], [12], [16], [17]. If the inducible release of sJAM-A under pro-inflammatory conditions really serves to limit leukocyte extravasation *in vivo* it is surprising that an EC type known for its strong barrier properties does not exhibit this inflammation-limiting feature while at least *in vitro* JAM-A functions allowing leukocyte extravasation, i.e. redistribution to the cell surface under inflammatory conditions, seem to function like in other vascular beds.

In summary, we demonstrated that the regulation of JAM-A cell surface expression and the release of sJAM-A by both primary and immortalized human brain endothelium share some features with EC types from other parts of the body but also show unique properties of brain EC, not described in other EC types so far. The non-inducibility of sJAM-A release by brain EC might reflect their special role in the protection of the CNS compartment.

## Supporting Information

Table S1 Clinical characteristics of multiple sclerosis patients. The table provides a comparison between stable and active patients concerning age, gender, disease duration and Expanded Disability Status Scale (EDSS), reflecting the degree of disability.
(0.03 MB DOC)

Table S2 Clinical characteristics of ischemic stroke patients. The table lists age, gender, NIH Stroke Scale (NIHSS) at admission, infarct size, type of infarct according to the TOAST criteria, time from clinical onset to first blood withdrawal and whether or not systemic thrombolysis was performed for all patients taking part in this study.
(0.05 MB DOC)
