# Supplementary material for: Evaluation of Soluble Junctional Adhesion Molecule-A as a Biomarker of Human Brain Endothelial Barrier Breakdown
Source: PLoS One. 2010 Oct 21;5(10):e13568. doi: 10.1371/journal.pone.0013568 (PMC2958838; doi:10.1371/journal.pone.0013568)
Supplement: Table S1 — Clinical characteristics of multiple sclerosis patients. The table provides a comparison between stable and active patients concerning age, gender, disease duration and Expanded Disability Status Scale (EDSS), reflecting the degree of disability. (0.03 MB DOC) [file pone.0013568.s001.doc]

**Table S1. Clinical characteristics of multiple sclerosis patients.**

|  | Stable  (n=45) | Acute relapse (n=14) | p value |
| --- | --- | --- | --- |
| Mean age (years)  (SD, range) | 40.0  (11.7, 19-68) | 41.7  (11.3, 17-64) | 0.48 |
| Female patients (%)  (n) | 69  (31) | 71  (10) | 0.98 |
| Mean disease duration (years)  (SD, range) | 9.4  (9.6, 1-46) | 9.6  (6.1, 1-19) | 0.52 |
| Mean EDSS  (SD, range) | 2.0  (1.3, 0-6.0) | 3.8  (2.1, 1.0-7.0) | <0.01 |

EDSS, Expanded Disability Status Scale
