# Supplementary material for: Evaluation of Soluble Junctional Adhesion Molecule-A as a Biomarker of Human Brain Endothelial Barrier Breakdown
Source: PLoS One. 2010 Oct 21;5(10):e13568. doi: 10.1371/journal.pone.0013568 (PMC2958838; doi:10.1371/journal.pone.0013568)
Supplement: Table S2 — Clinical characteristics of ischemic stroke patients. The table lists age, gender, NIH Stroke Scale (NIHSS) at admission, infarct size, type of infarct according to the TOAST criteria, time from clinical onset to first blood withdrawal and whether or not systemic thrombolysis was performed for all patients taking part in this study. (0.05 MB DOC) [file pone.0013568.s002.doc]

**Table S2. Clinical characteristics of ischemic stroke patients.**

| Patient  Number | Age  (years) | Gender | NIHSS | Infarct  size | TOAST | Hours to first blood withdrawal | rtPA |
| --- | --- | --- | --- | --- | --- | --- | --- |
| 1 | 84 | female | 18 | 3 | 2 | 3 | yes |
| 2 | 54 | male | 21 | 3 | 4 | 2 | yes |
| 3 | 81 | female | 17 | 2 | 2 | 1 | yes |
| 4 | 57 | female | 6 | 1 | 1 | 3 | yes |
| 5 | 79 | male | 21 | 3 | 2 | 3 | yes |
| 6 | 92 | male | 17 | 2 | 2 | 2 | yes |
| 7 | 86 | female | 15 | 1 | 2 | 1 | no |
| 8 | 51 | male | 8 | 1 | 2 | 2 | yes |
| 9 | 73 | male | 2 | 4 | 2 | 2 | no |
| 10 | 81 | male | 2 | 4 | 1 | 3 | no |
| 11 | 77 | male | 16 | 2 | 2 | 2 | yes |
| 12 | 86 | female | 21 | 3 | 2 | 3 | no |
| 13 | 78 | female | 19 | 1 | 1 | 2 | no |

Infarct size

1) <1/3, 2) 1/3-2/3, 3) >2/3 middle cerebral artery territory, 4) posterior circulation

TOAST

1) Large vessel, 2) cardio-embolic, 3) small vessel, 4) unknown/other

rtPA, recombinant tissue plasminogen activator
